# Virulence assessment of six major pathogenic *Candida* species in the mouse model of invasive candidiasis caused by fungal translocation

**DOI:** 10.1038/s41598-020-60792-y

**Published:** 2020-03-02

**Authors:** Tatsuro Hirayama, Taiga Miyazaki, Yuya Ito, Megumi Wakayama, Kazutoshi Shibuya, Kohei Yamashita, Takahiro Takazono, Tomomi Saijo, Shintaro Shimamura, Kazuko Yamamoto, Yoshifumi Imamura, Koichi Izumikawa, Katsunori Yanagihara, Shigeru Kohno, Hiroshi Mukae

**Affiliations:** 1 Department of Respiratory Medicine, Nagasaki University Graduate School of Biomedical Sciences, Nagasaki, Japan; 2 Department of Respiratory Medicine, Nagasaki University Hospital, Nagasaki, Japan; 3 Department of Infectious Diseases, Nagasaki University Graduate School of Biomedical Sciences, Nagasaki, Japan; 4 Department of Surgical Pathology, Toho University School of Medicine, Tokyo, Japan; 5 Department of Laboratory Medicine, Nagasaki University Hospital, Nagasaki, Japan

**Keywords:** Fungal host response, Fungal pathogenesis

## Abstract

Gastrointestinal colonization has been considered as the primary source of candidaemia; however, few established mouse models are available that mimic this infection route. We therefore developed a reproducible mouse model of invasive candidiasis initiated by fungal translocation and compared the virulence of six major pathogenic *Candida* species. The mice were fed a low-protein diet and then inoculated intragastrically with *Candida* cells. Oral antibiotics and cyclophosphamide were then administered to facilitate colonization and subsequent dissemination of *Candida* cells. Mice infected with *Candida albicans* and *Candida tropicalis* exhibited higher mortality than mice infected with the other four species. Among the less virulent species, stool titres of *Candida glabrata* and *Candida parapsilosis* were higher than those of *Candida krusei* and *Candida guilliermondii*. The fungal burdens of *C. parapsilosis* and *C. krusei* in the livers and kidneys were significantly greater than those of *C. guilliermondii*. Histopathologically, *C. albicans* demonstrated the highest pathogenicity to invade into gut mucosa and liver tissues causing marked necrosis. Overall, this model allowed analysis of the virulence traits of *Candida* strains in individual mice including colonization in the gut, penetration into intestinal mucosa, invasion into blood vessels, and the subsequent dissemination leading to lethal infections.

## Introduction

*Candida* species are the fourth leading cause of nosocomial bloodstream infections in the United States^1^. *Candida albicans, Candida glabrata*, *Candida parapsilosis*, *Candida tropicalis*, *Candida krusei*, and *Candida guilliermondii* comprise the main pathogens responsible for invasive candidiasis^2^, accounting for 93% of cases according to results from the ARTEMS DISK Global Antifungal Surveillance Study^3^, with *C. albicans* being the most frequently isolated species^2^.

*Candida* species represent ubiquitous commensal yeasts that constitute part of the normal human skin and gut microbiota. *Candida* species can be detected on the mucosal surfaces of approximately 50–70% of healthy humans^4^. *Candida* colonization is regarded as a prerequisite for endogenous infection, with the gut serving as an important source in the development of candidaemia^5^. The major pathogenetic mechanisms of invasive candidiasis include (1) disruption of the normal gastrointestinal microbiota (e.g. use of broad-spectrum antibiotics), which allows overgrowth of *Candida* species in the gut; (2) damage to the intestinal mucosal barrier (e.g. anticancer chemotherapy), which allows direct invasion of *Candida* cells into the bloodstream and abdominal cavity; and (3) impairment of the host immune response (e.g. neutropenia), which allows overgrowth of *Candida* cells and dissemination into the bloodstream and subsequent organs, leading to deep-seated infections in various organs^4^. However, to date most murine models of candidaemia have been generated by directly injecting a single large quantity of *Candida* cells via the tail-vein, whereas few established models have mimicked the step of translocation from the gut.

Notably, isolation rates of other *Candida* species aside from *C. albicans* from patients with candidaemia have been increasing in recent years^6^. Patient outcome may consequentially be related to several associated factors such as the antifungal susceptibility and virulence of each *Candida* species. For example, in randomized clinical trials^7^, *C. tropicalis* is associated with higher mortality rates than those of other *Candida* species whereas conversely *C. parapsilosis* is associated with lower mortality rates. To address these issues, in the present study we established a mouse model of disseminated candidiasis that developed through the translocation of *Candida* cells from the gut. We then applied this model for assessing the virulence of the six major *Candida* species, *C. albicans*, *C. glabrata*, *C. parapsilosis*, *C. tropicalis*, *C. krusei*, and *C. guilliermondii*.

## Results

### *In vitro* growth rates of *Candida* strains

The *in vitro* growth rates of the six *Candida* strains used in our *in vivo* experiments were compared in yeast peptone dextrose (YPD) broth at 30 °C (Supplementary Fig. S1). The growth curves for *C. albicans*, *C. glabrata*, *C. tropicalis*, and *C. krusei* were superimposable over the first 4 to 8 h. The growth rates of *C. parapsilosis* and *C. guilliermondii* during the logarithmic phase were slightly lower than those of the other four species. The doubling time for each strain was 120 min for *C. albicans*, 103 min for *C. glabrata*, 138 min for *C. parapsilosis*, 115 min for *C. tropicalis*, 109 min for *C. krusei*, and 142 min for *C. guilliermondii*.

### Development of a murine model of gut-disseminated invasive candidiasis

**Figure 1:**
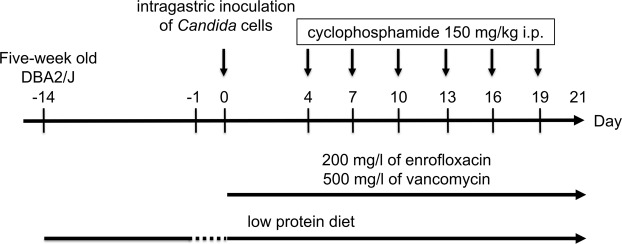
Schematic protocol of the gut-disseminated murine model of candidaemia. Five-week-old female DBA2/J mice were infected intragastrically with 0.2 ml of *Candida* cells (5 × 10^6^ CFU/ml) on day 0 following 14 d of a low protein diet and 24 h of food deprivation. The mice were provided sterile water containing 200 mg/l of enrofloxacin and 500 mg/l of vancomycin from the time of inoculation until the end of the experiment. Mice were treated intraperitoneally (i.p.) with cyclophosphamide (150 mg/kg of body weight) on days 4, 7, 10, 13, 16, and 19 post-infection.

Figure 1 shows the protocol used for development of the murine model of invasive candidiasis disseminated from the gut. To determine the best conditions for assessing colonization of *Candida* in the gut and its subsequent dissemination, we first developed mouse models using intragastric inoculation of the *C. albicans* wild-type strain ATCC MYA-2876 (SC5314) or *C. glabrata* wild-type strain ATCC 2001 (CBS138). Prior to infection, mice were fed a low protein diet to thin the mucosa of the small intestine and colon^8^, which predisposed to *Candida* colonization in the gut. Several factors affecting colonization and dissemination were analysed. In our preliminary studies, the DBA2/J mouse strain was more suitable for the translocation study than BALB/c mice because dissemination of *Candida* cells in DBA2/J mice was more pronounced than that in the BALB/c strain (Supplementary Fig. S2a,b). In addition, at three weeks of age, DBA2/J mice exhibited higher mortality rates and larger variations in the survival curve than those of five-week-old mice when infected with *C. glabrata* (Supplementary Fig. S2c). We further determined that the use of antibiotics was necessary during model development, as mice inoculated with *C. glabrata* without antibiotics exhibited higher mortality rates than those observed with concomitant antibiotics administration, which was likely due to the occurrence of bacterial translocation (Supplementary Fig. S2d).

A single inoculation of a *C. albicans* suspension was sufficient to induce colonization in the gut with subsequent dissemination, because fungal colonization levels in the stool, fungal burden in the liver, and survival rates were comparable between groups receiving a single inoculation and three consecutive inoculations (Supplementary Fig. S3a). We also determined no statistically significant differences in either the degree of fungal colonization, fungal burden in the liver, or survival rates among mice infected with a single 0.2 ml inoculation of cell suspension containing 5 × 10^6^, 5 × 10^7^, or 5 × 10^8^ colony forming units (CFU)/ml (Supplementary Fig. S3b). The level of fungal colonization in the stool reached a state of saturation at 6 d post-infection, as no change was observed until 8 d post-infection.

The administration of 150 mg/kg cyclophosphamide every three days was determined as a suitable dose/schedule to assess the virulence of different *Candida* species in the present study because uninfected mice receiving consecutive five days injections of 150 mg/kg cyclophosphamide, the schedule utilised in a previous study^9^, exhibited higher mortality than that observed upon injection every three days of 150 mg/kg cyclophosphamide (Supplementary Fig. S4a), suggesting that the mice died consequent to cyclophosphamide toxicity. Notably, the mice inoculated with *C. albicans* did not die following a single injection of 150 mg/kg cyclophosphamide (Supplementary Fig. S4b). However, injections of 75 mg/kg cyclophosphamide every three days did not elicit systemic spread of *C. glabrata* from the gut (Supplementary Fig. S4c,d).

Based on the results of these preliminary experiments, we established the protocol for the mouse model of invasive candidiasis caused by fungal translocation (Fig. 1). Using this protocol, *C. albicans* was first recovered from the liver at 8 d post-infection albeit not from the kidney, spleen, or blood at this time. No *Candida* cells were cultured at 21 d post-infection from stools and organs of mice administered saline without *Candida* at day 0.

### Comparison of *Candida* species virulence in a murine model of gut-disseminated invasive candidiasis

**Figure 2:**
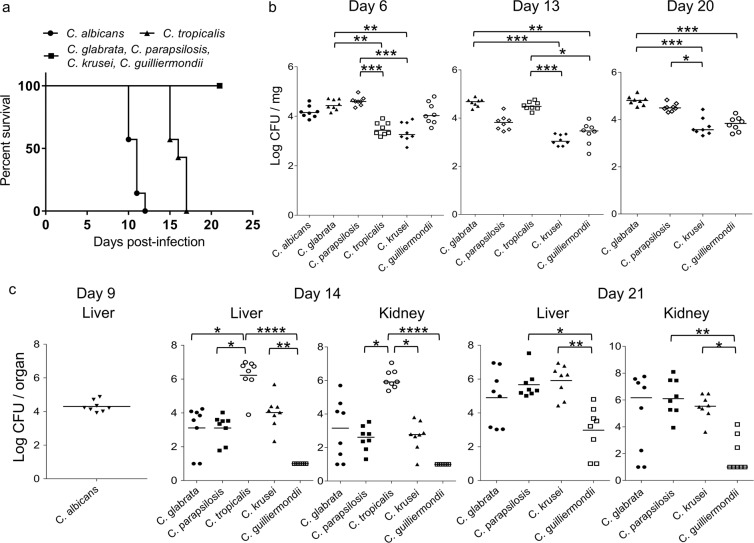
Comparison of *Candida* species virulence in a murine model of gut-disseminated invasive candidiasis. (**a**) Groups of mice (n = 7/group) were infected intragastrically with one of six *Candida* species on day 0 of the experiment and survival was monitored for 21 d post-infection. Kaplan–Meier curves were created and compared using the log-rank (Mantel–Cox) test. *P* = 0.0002 for *C. albicans* vs. *C. tropicalis*, *C. glabrata*, *C. parapsilosis*, *C. krusei*, and *C. guilliermondii. P* = 0.0003 for *C. tropicalis* vs. *C. glabrata*, *C. parapsilosis*, *C. krusei*, and *C. guilliermondii*. (**b**) Colonization in the intestinal tract is expressed as the log_10_ colony forming units (CFU)/mg of stool. Stool specimens were collected from groups of mice (n = 8/group) on the indicated days post-infection. The numbers of recovered CFU from the stools of individual mice are indicated in the plots. The geometric means are shown by the bars. Statistical analyses were performed using the Kruskal–Wallis test with Dunn’s multiple comparison post-test. Asterisks indicate statistically significant differences (***P < 0.001; **P < 0.01; *P < 0.05). (**c**) The development of disseminated candidiasis was evaluated as the log_10_ CFU/organ in the liver and kidneys. The livers and bilateral kidneys were removed from groups of mice (n = 8/group) on the indicated days post-infection. The numbers of recovered CFU from the livers and kidneys are indicated in the plots for individual mice. The geometric means are shown as bars. Statistical analyses were performed using the Kruskal–Wallis test with Dunn’s multiple comparison post-test. Asterisks indicate statistically significant differences (****P < 0.0001; **P < 0.01; *P < 0.05). These animal experiments were conducted on two separate occasions to ensure reproducibility. Representative data of two independent experiments are shown.

We next examined the virulence of six *Candida* species in mice infected intragastrically on day 0 with 0.2 ml of cell suspension (5 × 10^6^ CFU/ml). The mice infected with *C. albicans* and *C. tropicalis* exhibited 100% mortality by 12 and 17 d post-infection, respectively (Fig. 2a). The mice infected with the other four *Candida* species survived until the end of experiment (21 d post-infection). Mice infected with *C. albicans* exhibited higher mortality rates than those of mice infected with *C. tropicalis* (*P* = 0.0002) and mice infected with *C. tropicalis* exhibited higher mortality rates than those of mice infected with *C. glabrata*, *C. parapsilosis*, *C. krusei*, or *C. guilliermondii* (*P* = 0.0003).

Colonization level of *Candida* in the intestinal tract was expressed as log_10_ CFU/mg of the stool (Fig. 2b). Stool specimens were collected from groups of eight live mice at 6, 13, and 20 d post-infection. The CFU of *C. glabrata* and *C. parapsilosis* in the stool were higher than those of *C. tropicalis* and *C. krusei* with statistically significant differences at 6 d post-infection (*C. glabrata* vs. *C. tropicalis, P* = 0.003; *C. glabrata* vs. *C. krusei, P* = 0.002; *C. parapsilosis* vs. *C. tropicalis, P* = 0.0004; and *C. parapsilosis* vs. *C. krusei, P* = 0.0002). At 13 d post-infection, the colonization level of *C. tropicalis* had increased compared to that at day 6. In contrast, the colonization levels of *C. parapsilosis* and *C. guilliermondii* tended to decrease from day 6 to day 13 post-infection. Consequently, the CFU of *C. glabrata* and *C. tropicalis* in the stool were significantly higher than those of *C. krusei* and *C. guilliermondii* (*C. glabrata* vs. *C. krusei, P* < 0.0001; *C. glabrata* vs. *C. guilliermondii, P* = 0.002; *C. tropicalis* vs. *C. krusei, P* = 0.0003; and *C. tropicalis* vs. *C. guilliermondii, P* = 0.02). At 20 d post-infection, the CFU of *C. glabrata* in the stool were also significantly higher than those of *C. krusei* and *C. guilliermondii* (*C. glabrata* vs. *C. krusei, P* = 0.0001; *C. glabrata* vs. *C. guilliermondii, P* = 0.0005). Colonization levels of *C. parapsilosis* increased compared to those at 13 d post-infection, accordingly, the CFU of *C. parapsilosis* in the stool were significantly higher than those of *C. krusei* at 20 d post-infection (*C. parapsilosis* vs. *C. krusei, P* = 0.03).

To evaluate subsequent dissemination from the gut, fungal burden in the liver and kidneys was examined on 14 and 21 d post-infection (Fig. 2c). The mice infected with *C. albicans* were euthanized on day 9 as they were unable to survive to 14 d and the mice infected with *C. tropicalis* were euthanized on day 14 as they were unable to survive to 21 d post-infection (Fig. 2a). The log_10_ CFU/organ for the liver and kidneys were then calculated. At 9 d post-infection, *Candida* cells were isolated from the livers of all mice infected with *C. albicans* (8/8) albeit not from the kidneys (0/8). As shown in Fig. 2c, at 14 d post-infection, the fungal burden of *C. tropicalis* was greater than that of the other *Candida* species in the liver (*P* < 0.0001 for *C. tropicalis* vs. *C. guilliermondii*, *P* = 0.04 for *C. tropicalis* vs. *C. glabrata*, and *P* = 0.02 for *C. tropicalis* vs. *C. parapsilosis*) and in the kidneys (*P* < 0.0001 for *C. tropicalis* vs. *C. guilliermondii*, *P* = 0.04 for *C. tropicalis* vs. *C. parapsilosis*, and *P* = 0.049 for *C. tropicalis* vs. *C. krusei*). The fungal burden of *C. krusei* in the liver was significantly higher than that of *C. guilliermondii* (*P* = 0.005).

At 21 d post-infection, the numbers of *Candida* cells recovered from the livers and kidneys increased for all species compared to those at 14 d post-infection. *Candida* cells were isolated from the livers of all mice infected with *C. glabrata* (8/8), *C. parapsilosis* (8/8), and *C. krusei* (8/8). In contrast, the isolation ratio was lower at 75% in mice infected with *C. guilliermondii* (6/8). *Candida* cells were also recovered from the kidneys of 100% of mice infected with *C. parapsilosis* (8/8) and *C. krusei* (8/8) compared to 75% of mice infected with *C. glabrata* (6/8) and 38% of *C. guilliermondii-*infected animals (3/8). The fungal burden induced by *C. parapsilosis* and *C. krusei* was higher than that by *C. guilliermondii* (liver, *P* = 0.02 for *C. parapsilosis* vs. *C. guilliermondii* and *P* = 0.004 for *C. krusei* vs. *C. guilliermondii;* kidney, *P* = 0.005 for *C. parapsilosis* vs. *C. guilliermondii* and *P* = 0.04 for *C. krusei* vs. *C. guilliermondii*). The blood-culture positive ratios were as follows: 0/8 for *C. albicans* (day 9), 8/8 for *C. tropicalis* (day 14), 5/8 for *C. glabrata* (day 21), 8/8 for *C. parapsilosis* (day 21), 6/8 for *C. krusei* (day 21), and 2/8 for *C. guilliermondii* (day 21).

Because the results of the previous experiment were obtained with a single strain for each *Candida* species, we also conducted a second experiment using different wild-type strains with the same protocol. As a result, the mice infected with *C. albicans* and *C. tropicalis* exhibited 100% mortality by 17 and 15 d post-infection, respectively (Supplementary Fig. S5a). The mice infected with the other four *Candida* species survived until the end of the experiment (21 d post-infection). Mice infected with *C. tropicalis* exhibited higher mortality than that of mice infected with *C. albicans* (*P* = 0.014) and mice infected with *C. albicans* exhibited higher mortality than that of mice infected with *C. glabrata*, *C. parapsilosis*, *C. krusei*, or *C. guilliermondii* (*P* = 0.0002 each).

Colonization levels of *Candida* in the intestinal tract are shown in Supplementary Fig. S5b. The CFU of *C. albicans*, *C. glabrata*, *C. parapsilosis*, and *C. guilliermondii* in the stool were higher than those of *C. krusei* with statistically significant differences at 6 d post-infection (*C. albicans* vs. *C. krusei, P* = 0.0043; *C. glabrata* vs. *C. krusei, P* = 0.0003; *C. parapsilosis* vs. *C. krusei, P* = 0.008; and *C. guilliermondii* vs. *C. krusei, P* = 0.041). At 13 d post-infection, the CFU of *C. glabrata* in the stool were significantly higher than those of *C. krusei* and *C. guilliermondii* (*C. glabrata* vs. *C. krusei, P* = 0.028; *C. glabrata* vs. *C. guilliermondii, P* < 0.0001). At 20 d post-infection, the CFU of *C. glabrata* and *C. parapsilosis* in the stool were significantly higher than those of *C. guilliermondii* (*C. glabrata* vs. *C. guilliermondii, P* < 0.0001; *C. parapsilosis* vs. *C. guilliermondii, P* = 0.017).

Fungal burden in the liver and kidneys was also examined on 14 and 21 d post-infection (Supplementary Fig. S5c). At 14 d post-infection, the fungal burden induced by *C. krusei* was significantly higher than that by *C. guilliermondii* (liver, *P* < 0.0001; kidney, *P* = 0.0073). At 21 d post-infection, the fungal burdens induced by *C. glabrata* and *C. krusei* were significantly higher than that by *C. guilliermondii* (liver, *P* = 0.033 for *C. glabrata* vs. *C. guilliermondii* and *P* < 0.0001 for *C. krusei* vs. *C. guilliermondii;* kidney, *P* < 0.0001 for *C. glabrata* vs. *C. guilliermondii*).

### Histopathological evaluation

**Figure 3:**
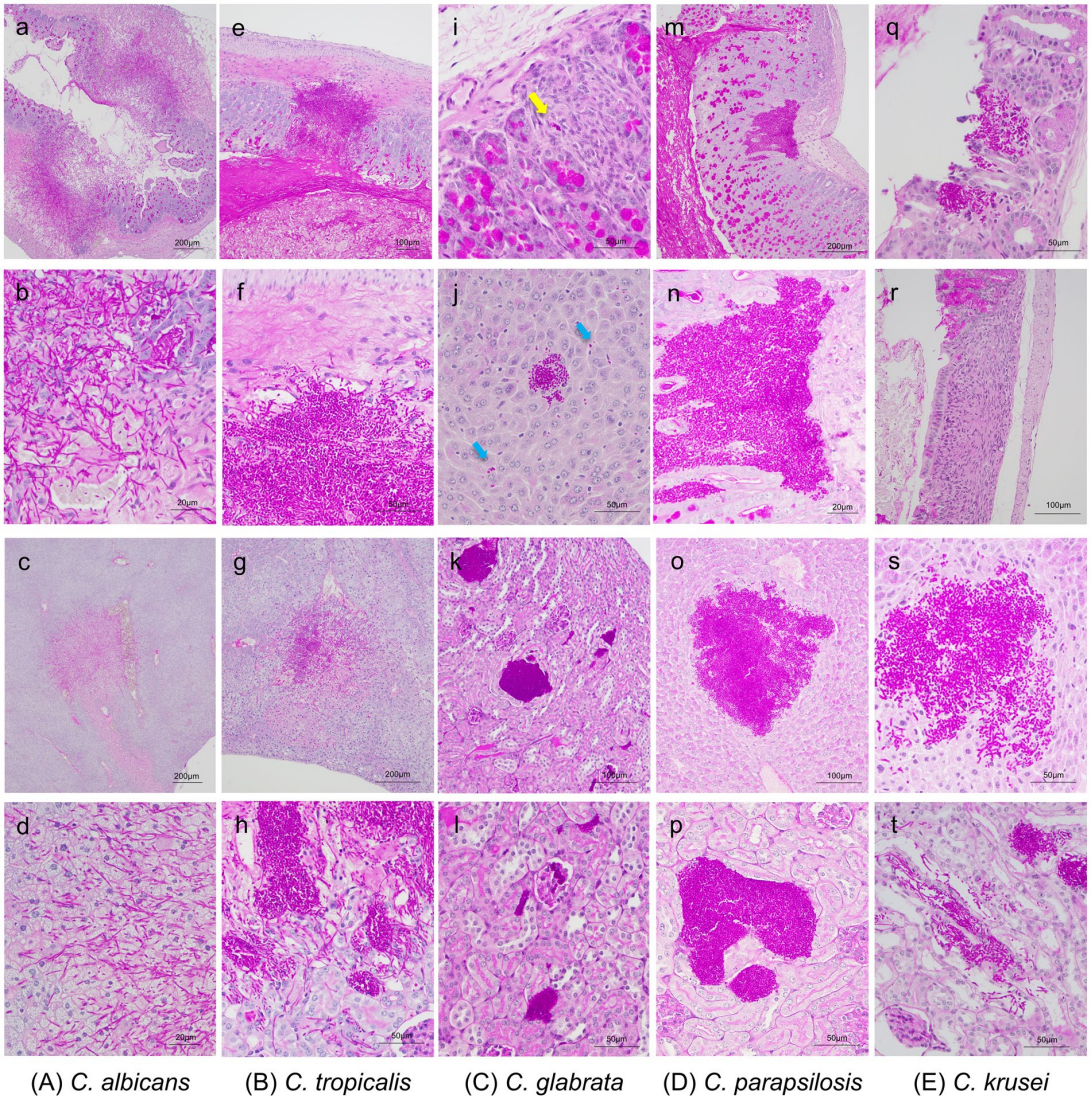
Histopathological examination using periodic acid-Schiff (PAS) staining. (**a**–**d**) Histopathological sections from DBA2/J mice infected with *Candida albicans*. *C. albicans* invaded into the gut mucosa and liver tissue resulting in marked necrosis. *C. albicans* was not identified in the kidney. (**a**) Cecum, magnification ×40; (**b**) cecum, magnification ×400; (**c**) liver, magnification ×40; or (**d**) liver, magnification ×400. (**e**–**h**) Histopathological sections from mice infected with *Candida tropicalis*. *C. tropicalis* invaded into the muscle layer of the colon. The majority of colonies were adjacent to blood vessels in the liver and were present in the renal pelvis and tubules in the kidney. (**e**) Colon, magnification ×100; (**f**) colon, magnification ×400; (**g**) liver, magnification ×100; and (**h**) kidney, magnification ×400. (**i**–**l**) Histopathological sections from mice infected with *Candida glabrata*. Only a few *C. glabrata* cells were observed in the gut (yellow arrow). *C. glabrata* was widely found in the liver along the sinusoid (blue arrows) with a few areas of accumulated yeast cells. The majority of colonies were located in the renal tubules and interstitium. (**i**) Colon, magnification ×400; (**j**) liver, magnification ×400; (**k**) kidney, magnification ×200; and (**l**) kidney, magnification ×400. (**m**–**p**) Histopathological sections from mice infected with *Candida parapsilosis*. *C. parapsilosis* cells were found in the gut mucosa in the colon; however, they did not invade into the muscularis mucosae. The majority of colonies were located in the renal pelvis and tubules in the kidneys. (**m**) Colon, magnification ×100; (**n**) colon, magnification ×400; (**o**) liver, magnification ×200; and (**p**) kidney, magnification ×400. (**q**–**t**) Histopathological sections from mice infected with *Candida krusei*. Only one location of accumulated *C. krusei* cells was detected in the colons of three mice (**q**). Several granulation tissues were observed in the colon (**r**). *C. krusei* presented as both yeast and hypha form in the liver and kidneys. (**q**) Colon, magnification ×100; (**r**) colon, magnification ×200; (**s**) liver, magnification ×400; and (**t**) kidney, magnification ×400.

The cecum, colon, liver, and bilateral kidneys of at least three mice infected with each of the *Candida* strains were also examined histopathologically (Fig. 3). *C. albicans* presented in both yeast and hyphal forms as having invaded into the gut mucosa of the cecum and colon, associated with considerable tissue necrosis at 9 d post-infection (Fig. 3a–d). Invasive growth of *C. albicans* and tissue necrosis were confirmed in the livers but were not identified in the kidneys. *C. tropicalis* also presented in both yeast and hyphal forms as having invaded into the muscle layer of the colon at 14 d post-infection (Fig. 3e–h). The invasive growth of *C. tropicalis* was observed in the liver and kidneys. The damage of surrounding tissues with *C. tropicalis* infection was less than that with *C. albicans*. Very few *C. glabrata* cells were detected in the gut mucosa at 21 d post-infection (Fig. 3i–l). A few aggregated *C. glabrata* cells were widely observed along the sinusoid in the liver. The majority of colonies were located in the renal tubules and interstitium, and some were also detected in the renal glomerules.

*C. parapsilosis* presented only in the yeast form and was found in the gut mucosa in the colon at 21 d post-infection; however, unlike *C. albicans* and *C. tropicalis, C. parapsilosis* did not invade into the muscularis mucosae (Fig. 3m–p). A single location of cell accumulation and several granulation tissues were observed in the colons of three mice infected with *C. krusei* at 21 d post-infection (Fig. 3q–t)*. C. parapsilosis* and *C. krusei* cells were observed in both the livers and kidneys of infected mice. *C. guilliermondii* was not pathologically identified in the gut mucosa, liver, or kidneys of any mice at 21 d post-infection. The location and morphology of *Candida* cells were revealed more clearly in high magnification images (Supplementary Fig. S6). Unlike the observations at 21 d post-infection, although the organs of mice infected with *C. glabrata*, *C. parapsilosis* and *C. krusei* were examined histopathologically at 14 d post-infection, *Candida* cells were not pathologically identified in the gut mucosa, liver, or kidneys of any mice at this point.

### Comparison of *Candida* species virulence in an intravenous injection model

**Figure 4:**
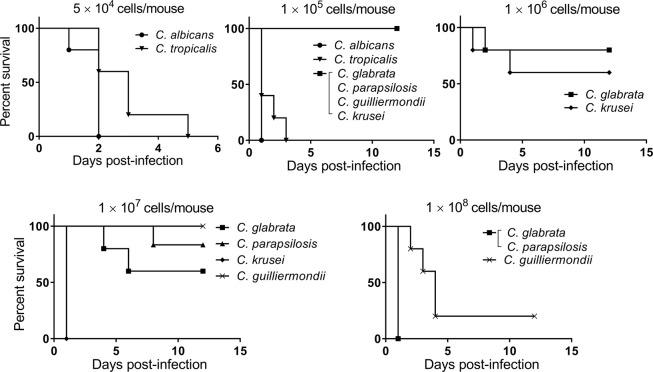
Survival study of mice inoculated intravenously with six *Candida* species. Groups of mice (n = 5/group) were intravenously infected with one of six *Candida* species on day 0 of the experiment and survival was monitored for 12 d post-infection. Kaplan–Meier curves were created and compared using the log-rank (Mantel–Cox) test. Upper left panel (5 × 10^4^ CFU/mouse): *P* = 0.04 for *C. albicans* vs. *C. tropicalis*; upper centre panel (1 × 10^5^ CFU/mouse): *P* = 0.003 for *C. albicans* vs. *C. glabrata*, *C. parapsilosis*, *C. krusei*, and *C. guilliermondii*; *P* = 0.002 for *C. tropicalis* vs. *C. glabrata*, *C. parapsilosis*, *C. krusei*, and *C. guilliermondii*; *P* = 0.13 for *C. albicans* vs. *C. tropicalis*; upper right panel (1 × 10^6^ CFU/mouse): *P* = 0.52 for *C. glabrata* vs. *C. krusei*; lower left panel (1 × 10^7^ CFU/mouse): *P* = 0.003 for *C. glabrata* vs. *C. krusei* and *C. krusei* vs. *C. guilliermondii*; *P* = 0.002 for *C. parapsilosis* vs. *C. krusei*; *P* = 0.34 for *C. glabrata* vs. *C. parapsilosis*; *P* = 0.13 for *C. glabrata* vs. *C. guilliermondii*; *P* = 0.36 for *C. parapsilosis* vs. *C. guilliermondii*; lower right panel (1 × 10^8^ CFU/mouse): *P* = 0.005 for *C. glabrata* vs. *C. guilliermondii* and *C. parapsilosis* vs. *C. guilliermondii*.

When mice were infected with *Candida* species by direct intravenous injection, the *C. albicans*-infected group showed the lowest survival rate followed by the *C. tropicalis*-infected and *C. krusei*-infected groups (Fig. 4). The mice infected with *C. krusei* exhibited higher mortality than those infected with *C. glabrata* when 1 × 10^7^ cells were injected; in contrast, no statistically significant difference was observed between the *C. krusei*-infected and *C. glabrata*-infected groups when 1 × 10^6^ cells were injected. The mice infected with 1 × 10^7^ cells of *C. krusei* appeared to die from embolism because *C. krusei* cells are larger than other *Candida* cells. The mice infected with *C. glabrata* and *C. parapsilosis* exhibited similar survival rates. *C. guilliermondii* demonstrated the lowest virulence in this experiment.

## Discussion

We established a murine model of candidaemia developing from the gut, which was useful to assess intestinal *Candida* colonization and subsequent dissemination to other organs including the liver and kidneys. Key risk factors for developing candidaemia in humans include neutropenia, mucositis, and the use of broad-spectrum antibiotics^10,11^. In order to mimic these pathological aspects, we utilised cyclophosphamide to induce neutropenia^12^, along with enrofloxacin and vancomycin to reduce endogenous murine gastrointestinal bacteria. We also fed the mice low protein diets, which, as previously reported, favour the implantation of *C. albicans* and *C. glabrata* in the gut^9,13^. In our model, the liver served as a preferential organ for infection because *C. albicans* invaded into the gut mucosa and liver before being disseminated to the kidneys (Figs. 2c and 3a–d), which was consistent with the conclusions of previous studies that the liver constituted a much more reliable organ for supporting infection and that *Candida* might translocate to the liver via the portal circulation^14,15^. Notably, in our model, *Candida* dissemination to the kidneys occurred through liver during the late stage of infection as the CFU of the liver and kidneys were comparable at 14 or 21 d post-infection (Fig. 2c).

With regard to gastrointestinal colonization, the mean CFU of *C. glabrata* and *C. parapsilosis* in the stool tended to be higher throughout the infection than those of *C. krusei* and *C. guilliermondii*. The *C. krusei* strain used in this study reached a saturated state with fewer cells than the other strains, as shown in the *in vitro* growth curve. Analysis of colonization levels in the gut suggested that clearance of *C. guilliermondii* cells was easier than that for the other species evaluated. The mean CFU of *C. tropicalis* in the gut were lower at 6 d post-infection than those of the other strains but increased by 13 d post-infection. This increase may have occurred owing to potentially weakened immune systems of the infected mice caused by severe candidaemia that resulted in death.

In the mortality experiments with our translocation model, the survival time of the *C. albicans*-infected group was shortest among the six *Candida* species. At 9 d post-infection, immediately before the mice infected with *C. albicans* died, *C. albicans* cells were recovered from the liver but not from the kidneys or blood. The mice infected with *C. albicans* were considered to have died consequent to intestinal necrosis because *C. albicans* invaded into the gut mucosa and caused marked necrosis prior to kidney dissemination. *C. albicans* is widely accepted as the most pathogenic *Candida* species^16,17^, which is consistent with our present results obtained from both the intravenous and translocation mouse models. The survival time of *C. tropicalis*-infected mice was shorter than those of mice infected with either *C. glabrata*, *C. parapsilosis*, *C. krusei*, or *C. guilliermondii*. Our results from this study were consistent with previous reports that *C. tropicalis* is associated with high mortality in clinical trials^7,18^ and high virulence in an intravenous injection mouse model^19^. The mice infected with *C. tropicalis* in the present study were considered to die as a result of candidaemia because a large number of *C. tropicalis* cells were isolated from the liver, kidneys, and blood at 14 d post-infection. Unlike the results following administration of a single *Candida* strain for each species, upon infection with a different wild-type strain, the *C. tropicalis*-infected group exhibited higher mortality rates than those of the *C. albicans*-infected group. Virulence levels of *C. albicans* and *C. tropicalis* were therefore strain-dependent albeit consistently higher than those of the other four *Candida* species.

A limitation of our mouse model is that the evaluation of virulence based solely on survival rate was not appropriate for less virulent species, with this metric only being applicable for *C. albicans* and *C. tropicalis*. However, fungal organ burden was useful for the evaluation of virulence of the other four *Candida* species.

A previous study showed that based on molecular methods, *C. albicans* blood isolates recovered from patients with candidaemia were often similar in identity to corresponding rectal isolates^20^. Furthermore, molecular typing studies support the concept that the gut is the main portal of entry for *C. glabrata* bloodstream infections^21^. Alternatively, a large majority of *C. parapsilosis* infections have been described as exogenous^20^ with the description of a possible endogenous source of *C. parapsilosis* candidaemia being limited to only a few studies^22^. Our results demonstrated that *C. parapsilosis*, in addition to *C. glabrata*, may have the potential to invade the bloodstream from the gut. Notably, data regarding the infection routes of *C. tropicalis* are both limited and contradictory. One report indicated that *C. tropicalis* disseminates systemically from the gastrointestinal tract more frequently than *C. albicans*^23^, whereas another reports *C. tropicalis* dissemination to be less frequent^24^. In the current study, *C. tropicalis* disseminated relatively frequently. These findings may be indicative of different virulence traits among the strains. To our knowledge, however, no prior studies have reported the primary infection routes of *C. krusei* or *C. guilliermondii*. The present study demonstrated that *C. krusei* was able to disseminate from the gastrointestinal tract. In comparison, *C. guilliermondii* presented the lowest frequency of dissemination, indicating that *C. guilliermondii* may be more restricted and less likely to invade the bloodstream from the gastrointestinal tract compared to other *Candida* strains.

The virulence of each species was also reflected in the histopathological examination. *C. albicans* invaded into the gut mucosa and liver tissues, causing marked necrosis. *C. tropicalis* also invaded into the muscle layer of the gut; however, it caused less damage to the surrounding tissues compared to that from *C. albicans*. *C. parapsilosis* appeared to be less pathogenic than *C. tropicalis* as *C. parapsilosis* did not invade into the muscle layer of the gut, even though it could be found in the gut mucosa. It has been reported that *C. glabrata* is able to survive and replicate within macrophages^25^. Monocytes may act as a vehicle for the dissemination of *C. glabrata* cells and provide the yeast protection from extracellular defences of the host^26^. In our model, although we found no evidence of *C. glabrata* directly invading the gut mucosa, numerous *C. glabrata* cells accumulated in the liver and kidneys, suggesting that the infection mechanisms of *C. glabrata* may differ from those of the other species, which directly invaded into the gut mucosa.

Several murine models of gastrointestinal-derived *C. albicans* or *C. glabrata* candidaemia have been reported previously^9,13–15,27^; however, few references exist to the colonization and dissemination capacity of several different *Candida* species examined within the same model. In addition, the present study using our newly-developed model was the first to show that pathological findings differed depending on the specific *Candida* species. This translocation model may therefore provide a useful tool to assess and evaluate the colonization and subsequent dissemination of *Candida* species *in vivo*. Furthermore, it allows the consideration of pathological findings regarding the invasion of gut mucosa that are not possible with conventional intravenous infection models. In addition, the present model is well suited for the assessment of immune responses in the gut activated by *Candida* infection, such as the secretion of chemokines, immunopathological markers, and immunocompetent cells, as these have been reported to differ depending on the species of *Candida*^26,28^. However, a limitation of our translocation model is that the process of establishment is more complicated and requires a longer time and higher costs than those required for the conventional candidaemia mouse model. In addition, a specific limitation of the present study is that the pathogenic analysis was limited to only two strains of each species. Therefore, broadly applying these data across all clinical isolates is not possible.

In conclusion, the current translocation model may provide a means to obtain better insight regarding the intricate relationships of *Candida* gut colonization and dissemination and facilitate the improvement of preventive therapy against invasive fungal infections. Previous studies have identified a variety of virulence factors of *Candida*, such as those that affect adhesion, filamentation, enzyme production, and stress responses, although their relative importance for each step of the infection process is not yet fully understood. Through the use of mutant strains or clinical isolates, the contribution of various factors to gut colonization, penetration into the wall of the intestinal tract, and subsequent invasion into the blood stream and dissemination might be assessed in individual mice. Our newly developed mouse model would therefore be useful for the further analysis of virulence factors and help define the pathogenic processes of invasive candidiasis.

## Materials and Methods

### *Candida* strains and culture conditions

A total of six strains of *Candida* species were used in the first experiment, *C. albicans* ATCC MYA-2876 (SC5314), *C. glabrata* ATCC 2001 (CBS138), *C. parapsilosis* ATCC 90018, *C. tropicalis* ATCC 750, *C. krusei* ATCC 6258, and *C. guilliermondii* ATCC 6260. The second experiment was conducted using the following strains; *C. albicans* ATCC 90028, *C. glabrata* ATCC 90030, *C. parapsilosis* ATCC 90018, *C. tropicalis* ATCC 42678, *C. krusei* ATCC 32196, and *C. guilliermondii* ATCC 9058. Monoclonal subcultures of each strain were stored at −80 °C until use. *Candida* cells were propagated in YPD medium [1% (wt/vol) yeast extract, 2% (wt/vol) peptone, and 2% (wt/vol) glucose] at 30 °C.

### Determination of *in vitro* growth rates

Fungal growth was compared between the six *Candida* species. The *Candida* strains were propagated in YPD broth and the cell concentrations adjusted to an initial optical density at 600 nm (OD_600_) of 0.1. The cultures were incubated in 100 ml flasks containing 40 ml YPD broth at 30 °C with shaking at 250 rpm for 48 h. Growth curves were plotted using the OD_600_ values measured during the culturing period. To calculate the doubling time of each strain, the OD_600_ readings were plotted against time on a semi-logarithmic graph.

### Animals and ethics statement

Specific-pathogen-free, seven-week-old female BALB/c mice were purchased from Japan SLC, Inc. (Shizuoka, Japan). Specific-pathogen-free, five-week-old female DBA2/J mice were purchased from Clea Japan, Inc. (Tokyo, Japan). All animals were housed in a pathogen-free environment in groups of five or six in filter-top cages with access to food and water *ad libitum*. All animal experiments were performed in full compliance with the Guide for the Care and Use of Laboratory Animals^29^ and all institutional regulations and guidelines for animal experimentation following review and approval by the Institutional Animal Care and Use Committee of Nagasaki University (protocol number 1407281164).

### Establishment of candidaemia mouse models

Five-week-old female DBA2/J mice were fed a low-protein diet containing 5% casein (Oriental Yeast Co, Ltd., Tokyo, Japan) for 14 d prior to infection. They were provided sterile water prior to infection and sterile water containing 200 mg/l of enrofloxacin and 500 mg/l of vancomycin following infection.

To prepare the inocula, the *Candida* strains were cultured in YPD broth at 30 °C and harvested during the logarithmic phase of growth, washed twice, and resuspended in sterile saline. The number of *Candida* cells was counted using a haemocytometer and the concentrations adjusted to 5 × 10^6^ cells/ml. The actual number of CFU in the inocula was confirmed by plating serial dilutions of the cell suspensions on YPD plates. After 24 h of food being withheld, the mice were infected intragastrically with 0.2 ml of cell suspensions (1 × 10^6^ CFU) on day 0 through a stainless-steel catheter with a blunt end (outer diameter, 0.92 mm; Fuchigami, Ltd., Fukuoka, Japan; Cat. no. 4202). Prior to infection, no fungus was identified from stool samples cultured on YPD agar with 200 mg/l of imipenem at 30 °C for 48 h. The mice were treated intraperitoneally with cyclophosphamide (Sigma-Aldrich Japan, Tokyo, Japan; C0768) at a concentration of 150 mg/kg of body weight on days 4, 7, 10, 13, 16, and 19 post-infection (Fig. 1).

### Evaluation of survival and fungal organ burden in mice inoculated intragastrically with six *Candida* species

Groups of DBA2/J mice (n = 7/group) were infected intragastrically with one of six *Candida* species on day 0 of the experiment and survival was monitored for 21 d post-infection. To evaluate fungal burden, the DBA2/J mice were euthanized 9 d post-inoculation of *C. albicans*, 14 d post-inoculation of *C. tropicalis*, *C. glabrata*, *C. parapsilosis*, *C. krusei*, and *C. guilliermondii*, and 21 d post-inoculation of *C. glabrata*, *C. parapsilosis*, *C. krusei* and *C. guilliermondii* (n = 8 for each *Candida* strain at each time point). No mice died prior to euthanasia in this experiment. Blood was collected from the inferior vena cava or by cardiac puncture under isoflurane anaesthesia and 200–500 µl of each blood sample was directly plated onto YPD plates with and without 200 mg/l of imipenem. An estimated concentration of >1 CFU/100 µl was considered to be blood-culture positive.

The livers and bilateral kidneys were aseptically removed and extensively homogenized in sterile saline. Stools from live mice were collected, weighed, and homogenized on days 6, 13, and 20 post-infection. Appropriate dilutions of organ and stool homogenates were plated onto YPD plates containing 200 mg/l of imipenem. Colonies were counted after 24–48 h of incubation at 30 °C. These animal experiments were conducted on two separate occasions to ensure reproducibility. Eight mice per group were used in each experiment. Representative data of two independent experiments are shown.

### Comparative virulence assessment of *Candida* species in intravenously-infected mice

The BALB/c mice were inoculated with each of the six *Candida* species via the lateral tail vein (n = 5 for each *Candida* strain). The mice were administered cyclophosphamide 4 d pre-infection (150 mg/kg), 1 d pre-infection (100 mg/kg), and 2 and 5 d post-infection (100 mg/kg)^30^. The infection doses of the *Candida* strains were 5 × 10^4^, 1 × 10^5^, 1 × 10^6^, 1 × 10^7^, and 1 × 10^8^ CFU/mouse in 0.2 ml sterile saline.

### Histopathological examination

The organs of at least three DBA/2J mice per *Candida* strain were examined histopathologically. Mice infected with *C. albicans*, *C. tropicalis*, and the other four *Candida* species were euthanized 9, 14, and 21 d post-infection, respectively. Cecum, colon, liver, and bilateral kidney specimens were obtained. No washing of the luminal contents was performed to avoid the extraction of the intraluminal microorganisms. All tissues were fixed in 10% buffered formalin and stained with haematoxylin-eosin or periodic acid Schiff using standard procedures.

### Statistical analysis

All statistical analyses were carried out using Prism 6.0 software (GraphPad Software, Inc., La Jolla, CA, USA) and the data were expressed as the means ± standard deviations (SD). Comparison of survival curves was performed using the log-rank (Mantel–Cox) test. Fungal burden in the organs and stool was analysed using the Mann–Whitney test for comparison of two groups and with the Kruskal–Wallis test and Dunn’s multiple comparison post-test for comparison of multiple groups. A *P* value < 0.05 was considered to be statistically significant.

## Supplementary information

Supplementary figure.
