# Supplementary material for: Virulence assessment of six major pathogenic Candida species in the mouse model of invasive candidiasis caused by fungal translocation
Source: Sci Rep. 2020 Mar 2;10:3814. doi: 10.1038/s41598-020-60792-y (PMC7052222; doi:10.1038/s41598-020-60792-y)
Supplement: Supplementary file 1 — Supplementary figure. [file 41598_2020_60792_MOESM1_ESM.pdf]

Virulence assessment of six major pathogenic *Candida* species in the mouse model of invasive candidiasis caused by fungal translocation

Tatsuro Hirayama, Taiga Miyazaki, Yuya Ito, Megumi Wakayama, Kazutoshi Shibuya, Kohei Yamashita, Takahiro Takazono, Tomomi Saijo, Shintaro Shimamura, Kazuko Yamamoto, Yoshifumi Imamura, Koichi Izumikawa, Katsunori Yanagihara, Shigeru Kohno, Hiroshi Mukae

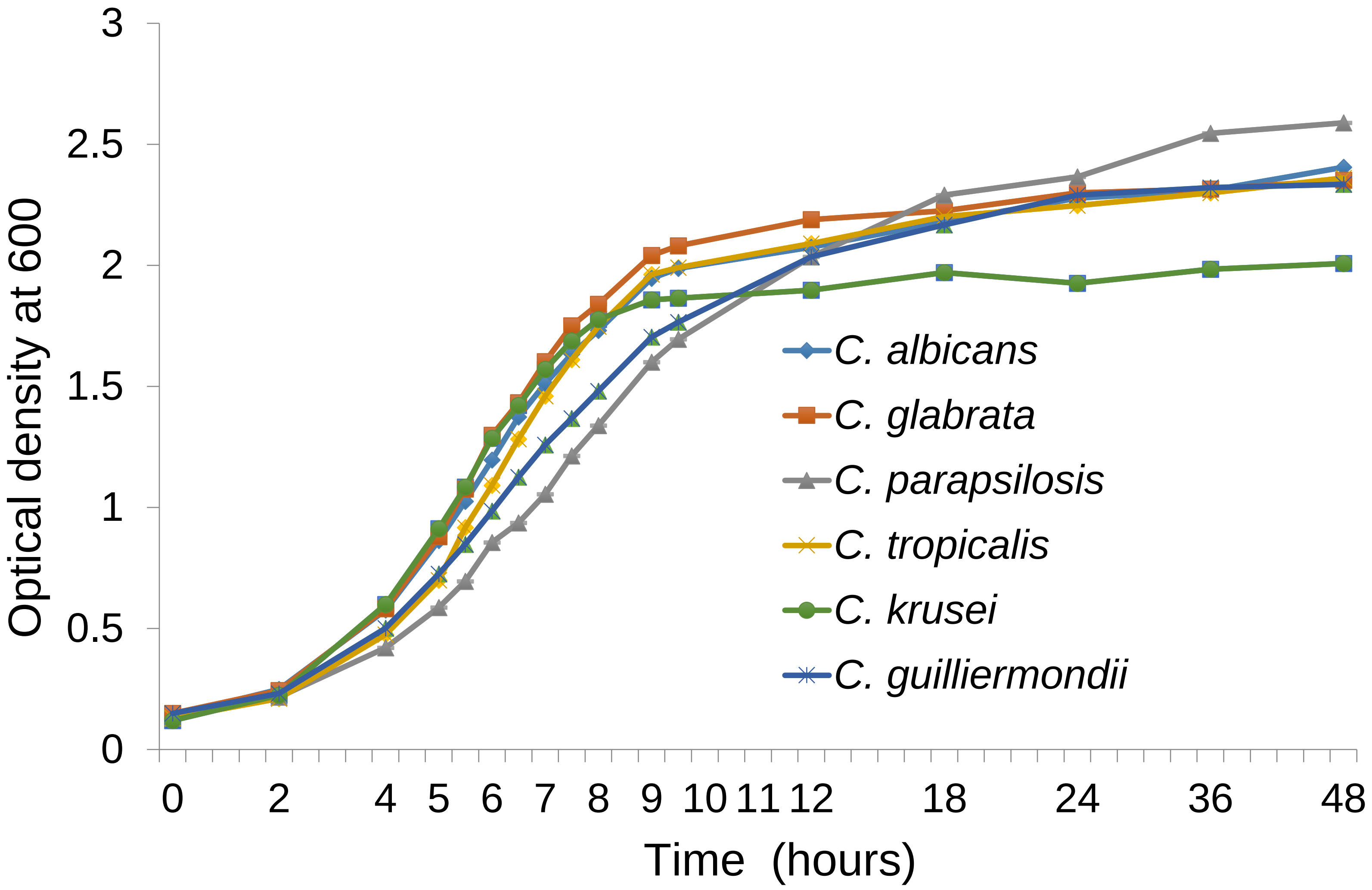

Supplementary Figure S1. Growth curves of *Candida* species cultured in YPD broth. The x axis represents a linear scale of time in hours and the y axis represents a scale of optical density (OD) as a measure of cell concentration. The cell growth demonstrated sigmoidal growth curves. The reproducibility of these results was confirmed on two separate occasions and representative data are shown.

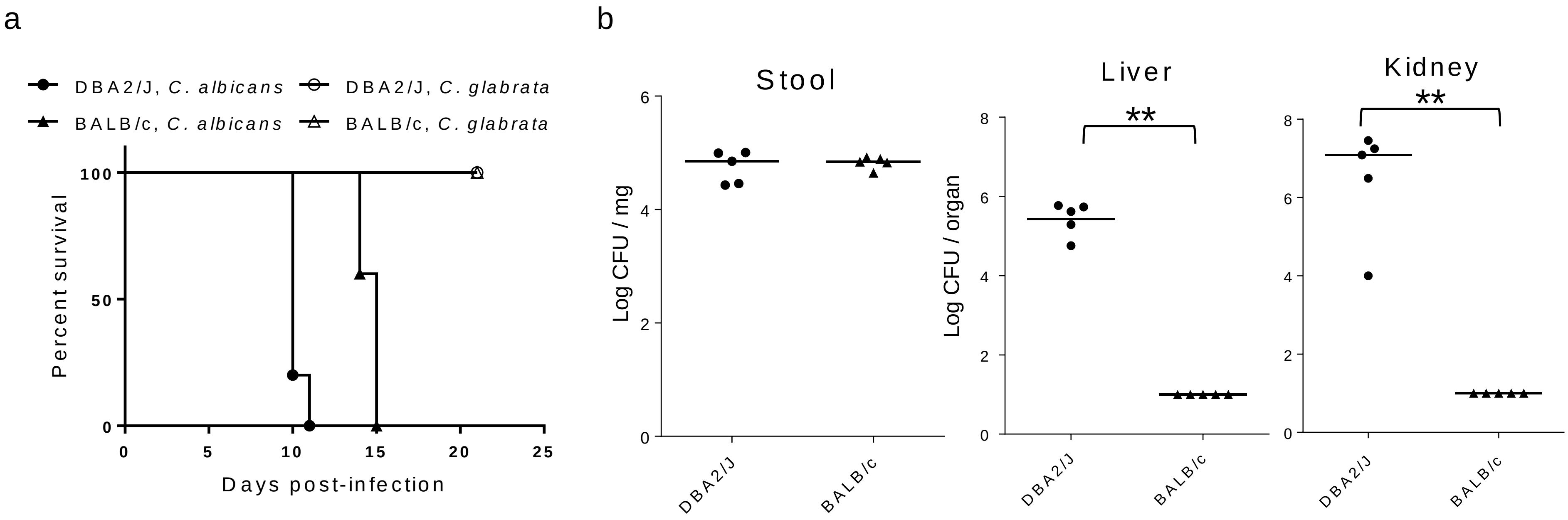

Supplementary Figure S2. Preliminary studies for development of a murine model of gut-disseminated invasive candidiasis infected with *Candida albicans* (SC5314) and *Candida glabrata* (CBS138), relating to mouse strain and age, and use of antibiotics.

(a) Groups of mice (n = 5/group) were infected intragastrically on day 0 of the experiment and survival was monitored for 21 d post-infection. The DBA2/J mice infected with *C. albicans* exhibited higher mortality than BALB/c (*P* = 0.0016, log-rank (Mantel–Cox) test).

(b) Groups of mice (n = 5/group) were infected with *C. glabrata* intragastrically on day 0. The development of disseminated candidiasis was evaluated as the log10 CFU/organ in the liver and kidneys and colonization in the intestinal tract is expressed as the log10 CFU/mg of stool at 21 d post-infection. *C. glabrata* disseminated more frequently in DBA2/J mice than in BALB/c mice (liver, *P* = 0.0079; kidney, *P* = 0.0079, Mann–Whitney test). No statistically significant differences were observed in the degree of fungal colonization between DBA2/J and BALB/c mice (*P* = 0.94, Mann–Whitney test).

c

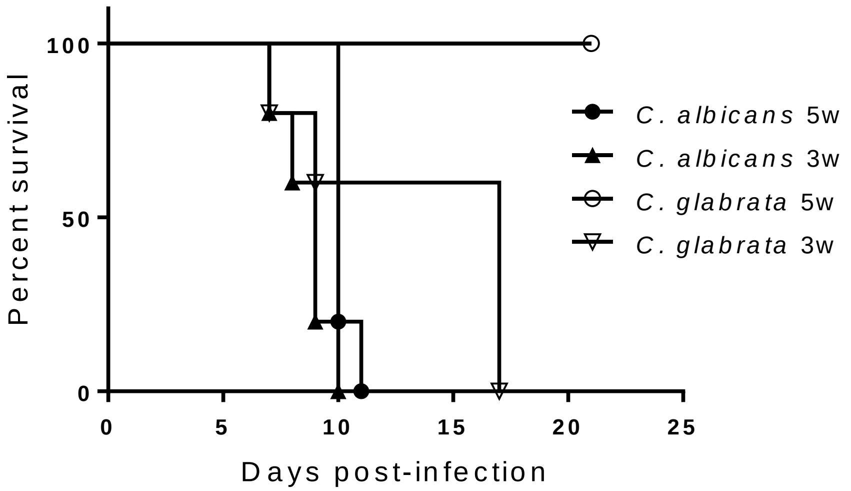

d

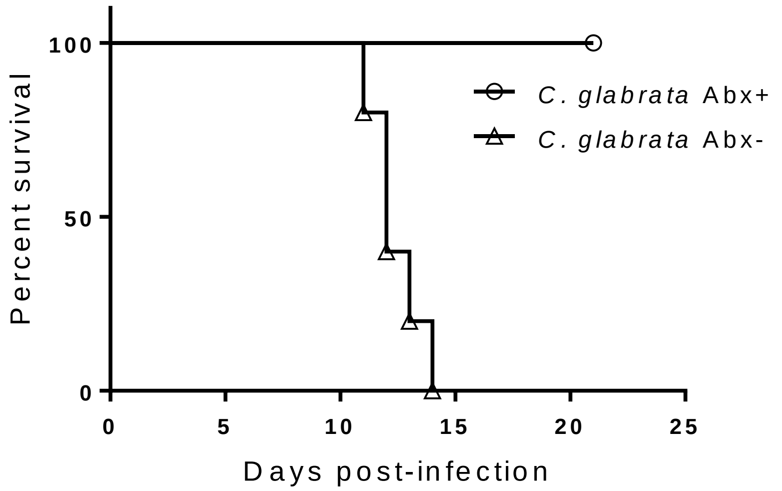

(**c**) Groups of three- (3w) and five-week-old (5w) mice (n = 5/group) were infected intragastrically. Three-week-old mice exhibited higher mortality than five-week old (*C. albicans*, *P* = 0.015; *C. glabrata*, *P* = 0.0034, log-rank (Mantel–Cox) test). Survival curves of the three-week-old mice exhibited large variation.

(**d**) Groups of mice (n = 5/group) were infected intragastrically with or without antibiotics (enrofloxacin and vancomycin). Mice inoculated with *C. glabrata* without antibiotics exhibited higher mortality rates than those receiving antibiotics (*P* = 0.0018, log-rank (Mantel–Cox) test). Abx indicates antibiotics (enrofloxacin and vancomycin).

a

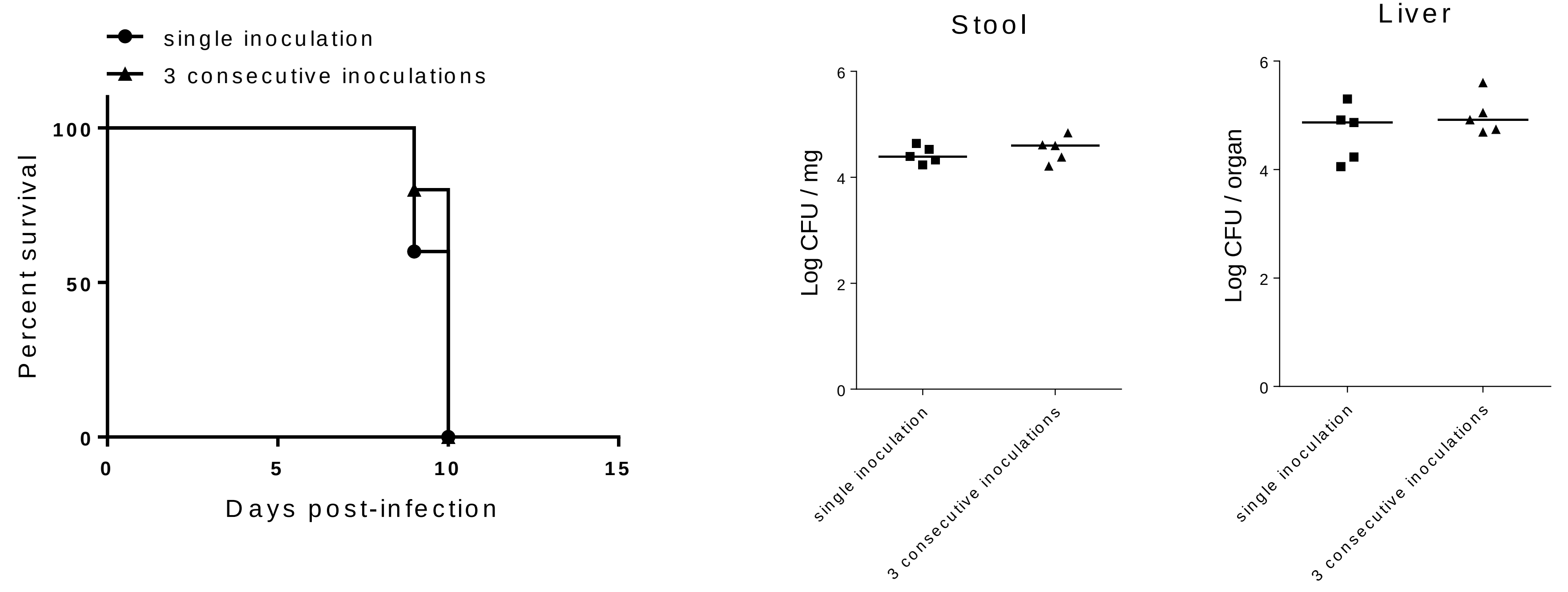

Supplementary Figure S3. Preliminary studies for development of a murine model of gut-disseminated invasive candidiasis infected with *Candida albicans* (SC5314), relating to the inoculation period and inoculum dosage.
(**a**) Groups of mice (n = 5/group) were infected with a single inoculation intragastrically on day 0 or with three days consecutive inoculation from day 0 to day 2 of the experiment and survival was monitored for 10 d post-infection. The development of disseminated candidiasis was evaluated as log10 CFU/organ in the liver and colonization in the intestinal tract is expressed as log10 CFU/mg of stool at 8 d post-infection. No statistically significant differences were observed in survival rates between the groups receiving single and three days consecutive inoculations (P = 0.51, log-rank (Mantel–Cox) test). No statistically significant differences were detected in fungal colonization levels in the stool and fungal burden in the liver at 8 d post-infection between the groups with single and three days consecutive inoculations (stool, P = 0.67; liver, P = 0.41, Mann–Whitney test).

b

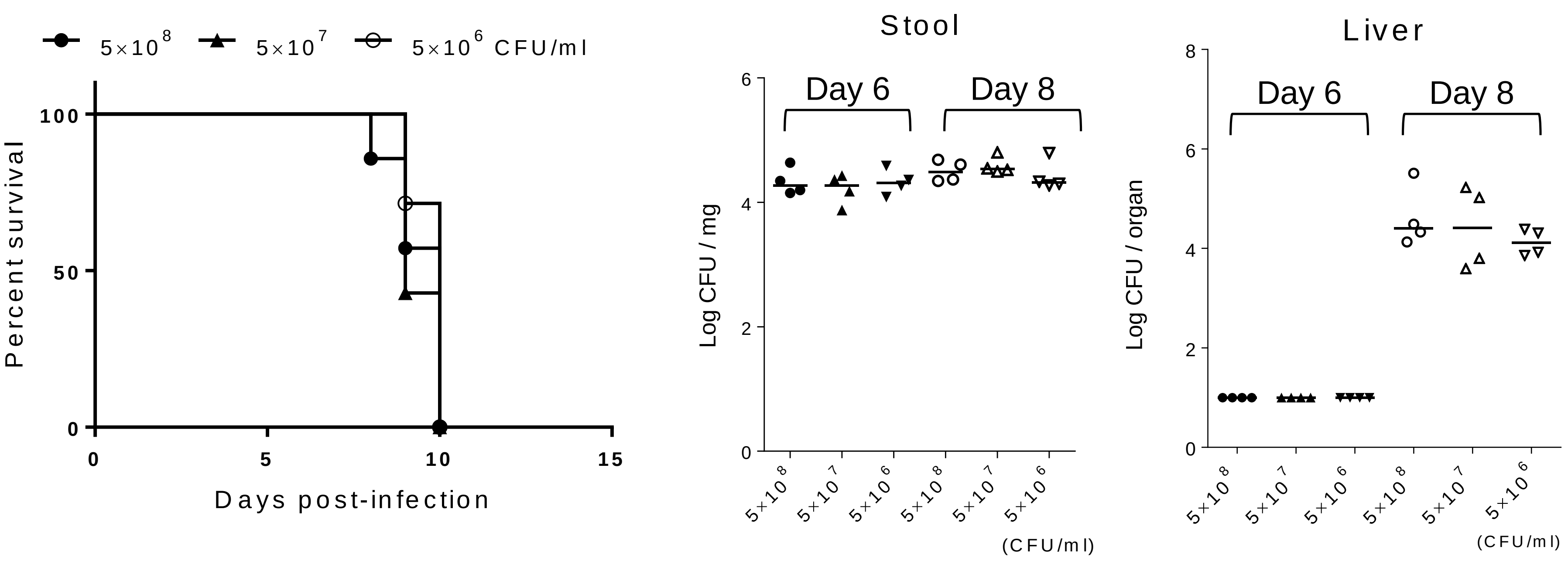

(**b**) Groups of mice (n = 4 or 5/group) were infected intragastrically with an inoculum of cell suspension containing 5×10^6, 5×10^7, or 5×10^8 CFU/ml on day 0 of the experiment and survival was monitored for 10 d post-infection. The development of disseminated candidiasis was evaluated as log10 CFU/organ in the liver and colonization in the intestinal tract is expressed as log10 CFU/mg of stool at 6 and 8 d post-infection. No statistically significant differences were observed in survival rates ($P = 0.30$ for 5×10^6 vs. 5×10^7; $P = 0.72$ for 5×10^6 vs. 5×10^8; $P = 0.80$ for 5×10^8 vs. 5×10^7, log-rank (Mantel–Cox) test). In addition, no statistically significant differences were observed in the degree of fungal colonization or the fungal burden in the liver at 6 and 8 d post-infection (Kruskal–Wallis test with Dunn’s multiple comparison post-test).

a

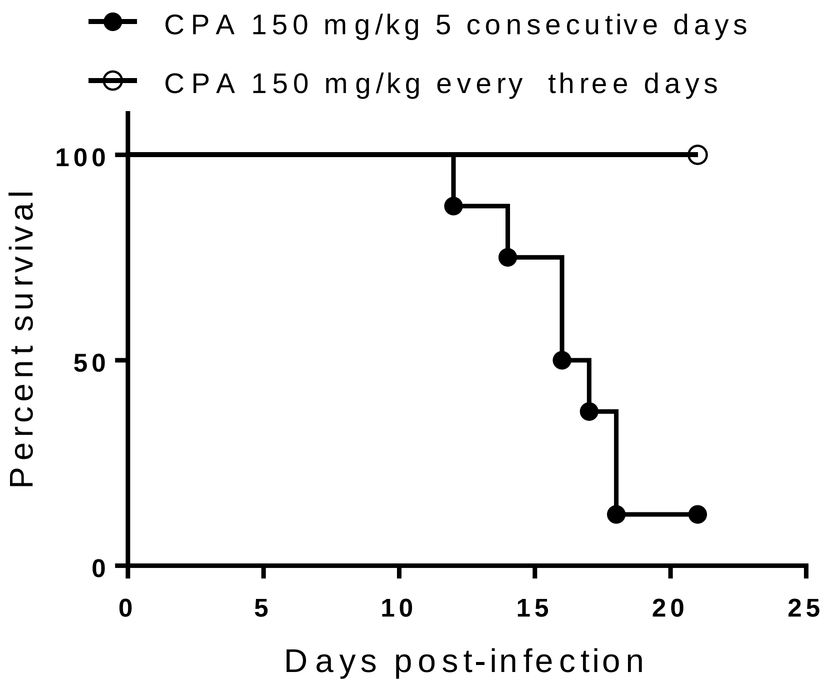

Supplementary Figure S4 . Preliminary studies for development of a murine model of gut-disseminated invasive candidiasis, relating to the dose of cyclophosphamide.
(**a**) Groups of uninfected mice (n = 8/group) were administered saline instead of a *Candida* suspension on day 0 of the experiment and survival was monitored for 21 days. The mice receiving consecutive five days injections of 150 mg/kg cyclophosphamide from day 4 exhibited higher mortality rates than those administered injections of 150 mg/kg cyclophosphamide every three days (*P* = 0.0005). Comparison of survival curves was performed using the log-rank (Mantel–Cox) test. CPA indicates cyclophosphamide.

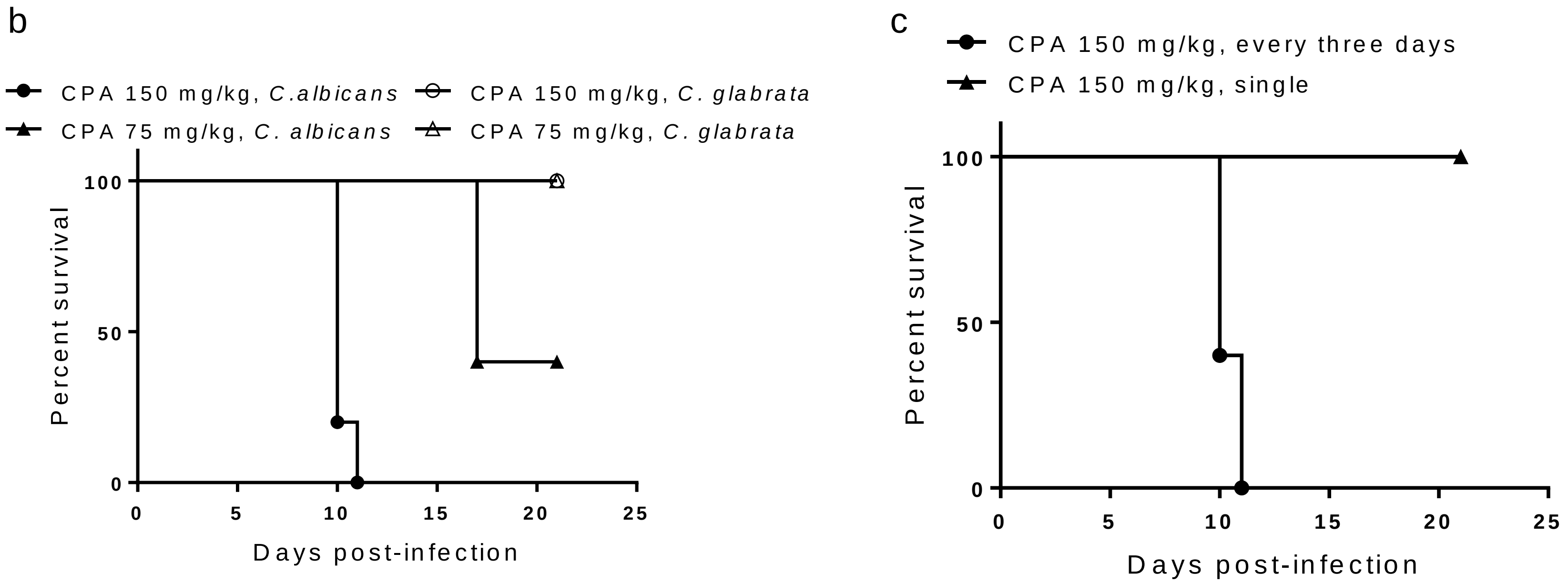

(**b**) Groups of mice (n = 5/group) were infected with *Candida albicans* intragastrically on day 0 of the experiment and survival was monitored for 21 d post-infection. No mortality was observed following a single injection of 150 mg/kg cyclophosphamide on 4 d post-infection. The mice receiving injections every three days of 150 mg/kg cyclophosphamide from 4 d post-infection exhibited higher mortality rates than those receiving a single injection of 150 mg/kg cyclophosphamide on 4 d post-infection. (*P* = 0.0023, log-rank (Mantel–Cox) test).

(**c**) Groups of mice (n = 5/group) were infected intragastrically on day 0 of the experiment and survival was monitored for 21 d post-infection. The development of disseminated candidiasis was evaluated as log10 CFU/organ in the liver and kidneys and colonization in the intestinal tract is expressed as log10 CFU/mg of stool at 21 d post-infection. The mice infected with *C. albicans* through injections every three days of 150 mg/kg cyclophosphamide exhibited higher mortality rates than those receiving injections every three days of 75 mg/kg cyclophosphamide (*P* = 0.0016, log-rank (Mantel–Cox) test).

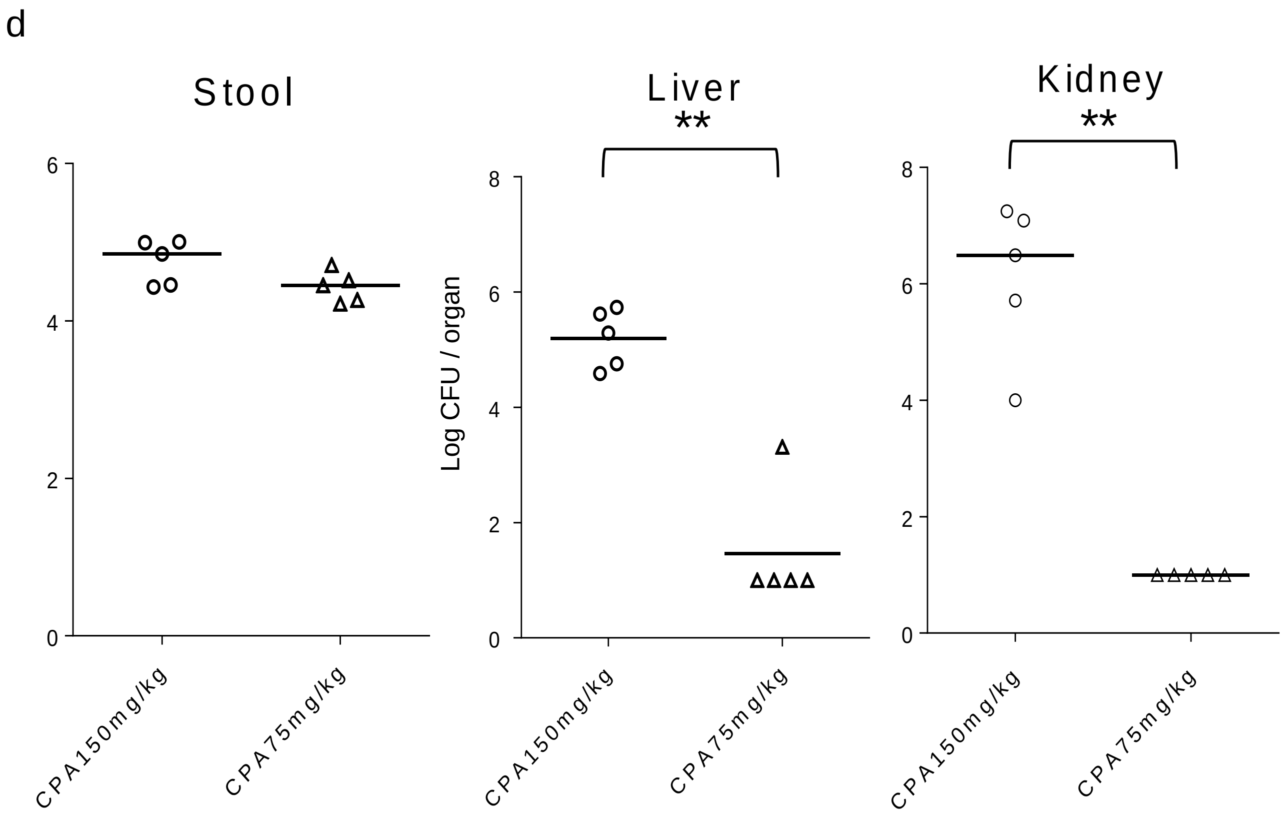

(**d**) Groups of mice (n = 5/group) were infected with *C. glabrata* intragastrically on day 0. The development of disseminated candidiasis was evaluated as log10 CFU/organ in the liver and kidneys and colonization in the intestinal tract is expressed as log10 CFU/mg of stool at 21 d post-infection. The fungal burdens of *Candida glabrata* following injections of 150 mg/kg cyclophosphamide in the liver and kidneys were statistically higher than those obtained following injections of 150 mg/kg cyclophosphamide (liver, $P = 0.0079$; kidney, $P = 0.0079$, Mann–Whitney test). No statistically significant differences were observed in fungal colonization levels in the stool ($P = 0.15$, Mann–Whitney test).

a

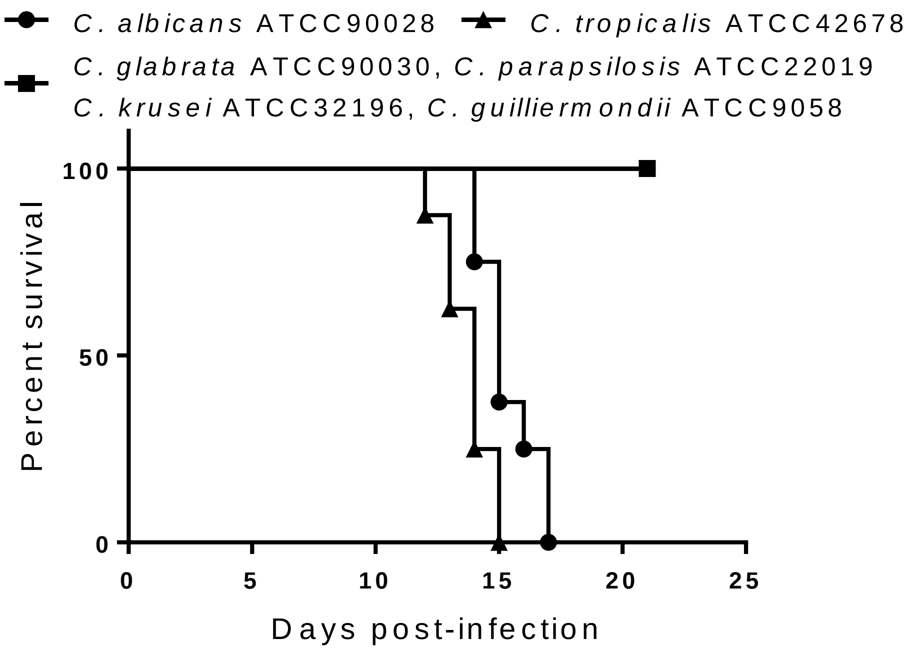

Supplementary Figure S5. The second experiment for comparison of *Candida* species virulence in a murine model of gut-disseminated invasive candidiasis. (**a**) Groups of mice (n = 8/group) were infected intragastrically with one of six *Candida* species on day 0 of the experiment and survival was monitored for 21 d post-infection. Kaplan–Meier curves were created and compared using the log-rank (Mantel–Cox) test. *P* = 0.014 for *Candida albicans* vs. *Candida tropicalis*. *P* = 0.0002 for *C. albicans* vs. *Candida glabrata*, *Candida parapsilosis*, *Candida krusei*, and *Candida guilliermondii. P* = 0.0002 for *C. tropicalis* vs. *C. glabrata*, *C. parapsilosis*, *C. krusei*, and *C. guilliermondii.*

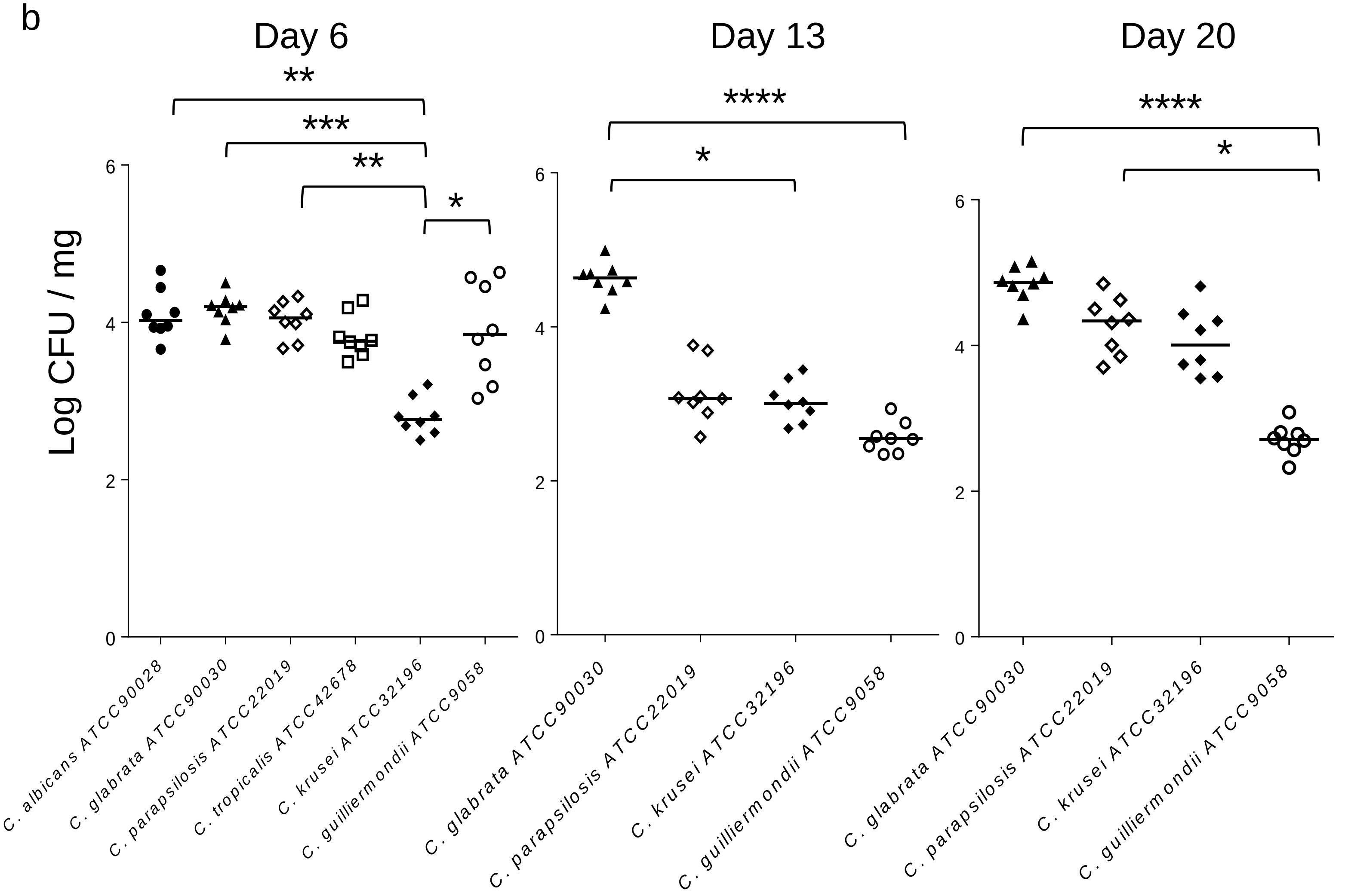

(**b**) Colonization in the intestinal tract is expressed as log10 CFU/mg of stool. Stool specimens were collected from groups of mice (n = 8/group) on the indicated days post-infection. The numbers of recovered CFU from the stool of individual mice are indicated in the plots. The geometric means are shown by the bars. Statistical analyses were performed using the Kruskal–Wallis test with Dunn's multiple comparison post-test. Asterisks indicate statistically significant differences (****P < 0.0001; ***P < 0.001; **P < 0.01; *P < 0.05).

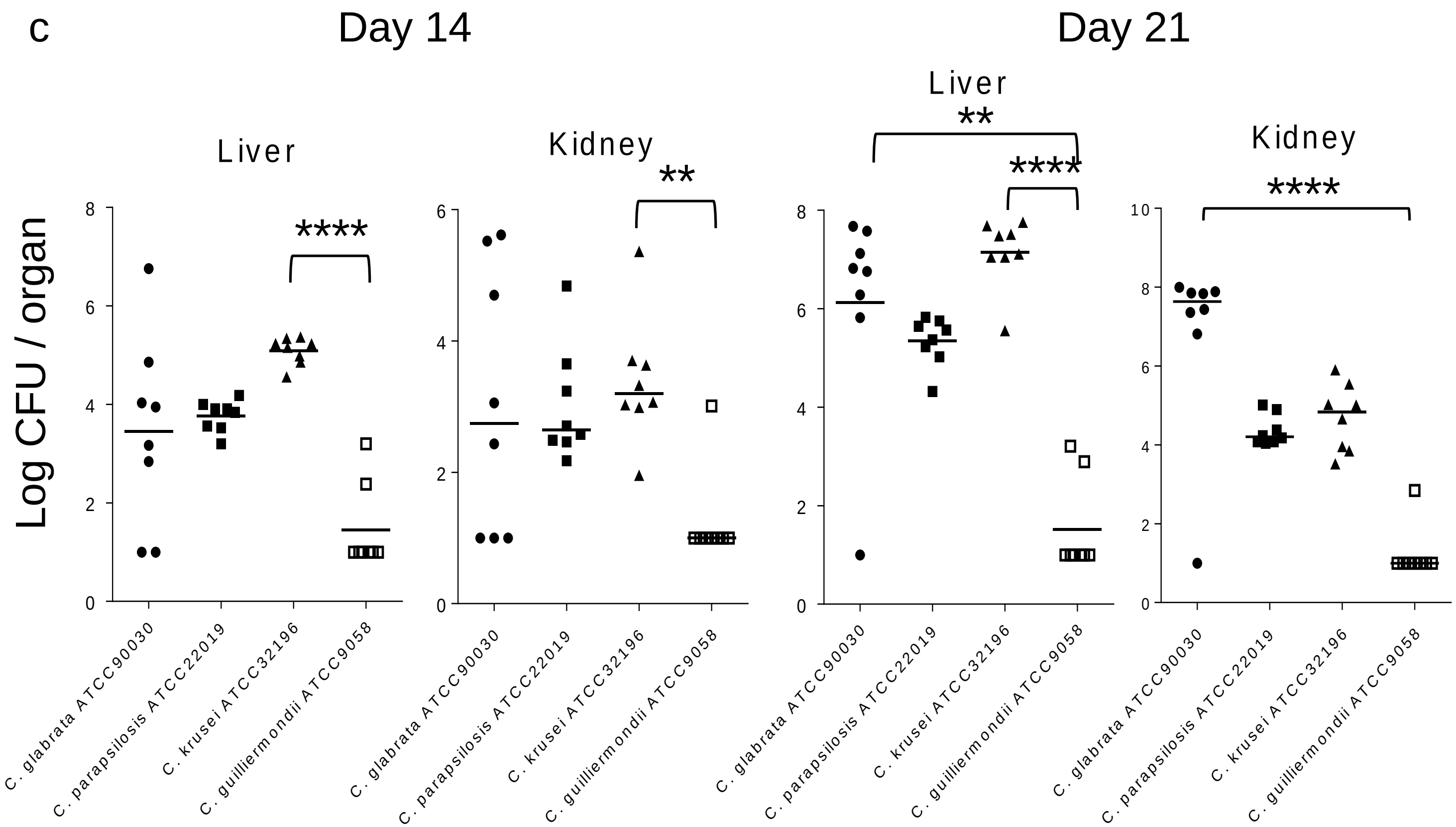

(**c**) The development of disseminated candidiasis was evaluated as log10 CFU/organ in the liver and kidneys. Livers and bilateral kidneys were removed from groups of mice (n = 8/group) on the indicated days post-infection. The numbers of recovered CFU from the livers and kidneys are indicated in the plots for individual mice. The geometric means are shown as bars. Statistical analyses were performed using the Kruskal–Wallis test with Dunn’s multiple comparison post-test. Asterisks indicate statistically significant differences (****$P < 0.0001$; **$P < 0.01$).

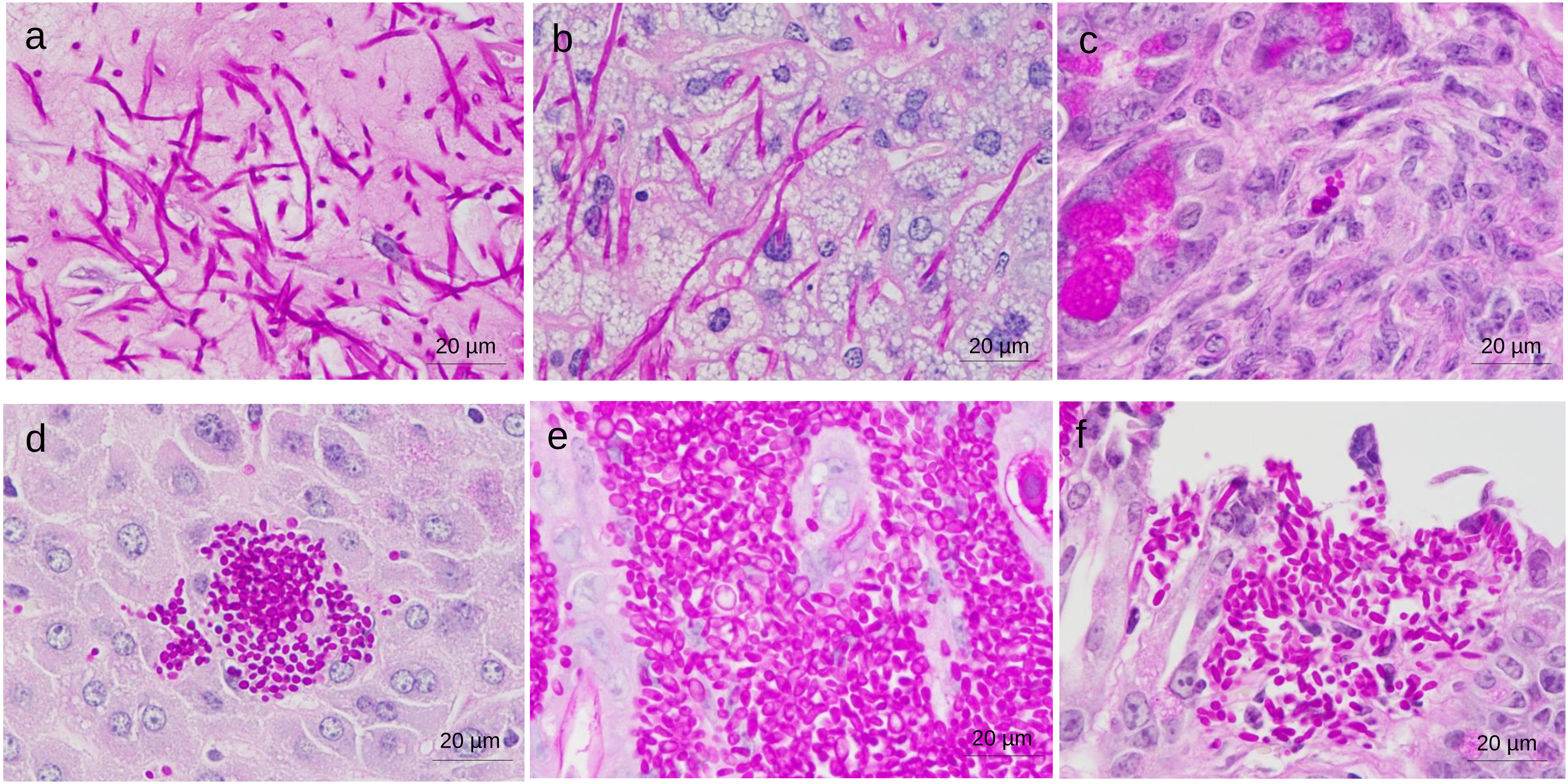

Supplementary Figure S6. High magnification (×1000) images of histopathological findings ascertained using periodic acid-Schiff (PAS) staining. (**a**) *Candida albicans*, cecum; (**b**) *C. albicans,* liver; (**c**) *Candida glabrata*, colon; (**d**) *C. glabrata*, liver; (**e**) *Candida parapsilosis*, colon; and (**f**) *Candida krusei*, colon.
